# Clearance In Vivo of Instilled [^3^H]Cholesterol from the Rat Lung

**DOI:** 10.1155/2010/965716

**Published:** 2010-02-10

**Authors:** Michael A. Wyder, Shannon M. Griffin, D. Nicole Worsham, Edna S. Kaneshiro

**Affiliations:** Department of Biological Sciences, University of Cincinnati, Cincinnati, OH 45221-0006, USA

## Abstract

Phospholipids and lung surfactant proteins are known to be recycled within the lung alveolus mainly by uptake into type II epithelial cells that secrete lipid-enriched lung surfactant. Dipalmitoyl phosphatidylcholine (DPPC) is the major component of lung surfactant lipids and cholesterol is the second most abundant. However, cholesterol turnover in vivo has not been measured and it is not known how long steroidal compounds persist in the lung in intact animals. Here we report on experiments in which radiolabeled cholesterol was instilled into the lungs of rats, then at various postinstillation periods, radioactive sterols in lavage fluid, and in postlavage whole lungs were measured in individual animals. Radioactive sterols in the lungs remained high for a week and were still detectable 46 days later. The clearance rate during the initial postinstillation week was approximately 10% per day. Both radioactive free and esterified sterols were recovered from bronchoalveolar lavage fluid and postlavage lungs.

## 1. Introduction

Lung surfactant is maintained at precise concentrations in the alveolar space by the regulation of synthesis and catabolism of surfactant components [1]. Furthermore, lung surfactant apoproteins and phospholipids are recycled within the mammalian lung [2–13], primarily by mechanisms involving alveolar epithelial type II pneumocyte uptake and secretion. Electron microscopic cytochemical and biochemical subcellular fractionation experiments demonstrate binding and uptake of labeled surfactant components by type II pneumocytes [5, 7, 9, 11, 12]. The labeled material becomes associated with intracellular organelles such as endosomes and multilamellar bodies and in various cytoplasmic subcellular fractions. Studies on recycling, clearance and lung compartmentalization of surfactant components were performed using in vitro, ex vivo, and in vivo studies [1, 5, 8, 11, 12]. Radiolabeled compounds or tagged material visualized by microscopy have been employed to follow the translocation of Surfactant Protein A (SP-A) and the dominant lung surfactant component dipalmitoyl phosphatidylcholine (DPPC). The majority of reports to date are on experiments in which the labeled surfactant compound is analyzed within minutes to hours after exposure to the label. For example, studies have been performed on perfused lung preparations using small animals [2, 4]. Also, a recent study involving gamma scintigraphy technology was employed in humans to follow deposition and clearance in vivo of aerosolized ^99m^Tc liposomes containing DPPC, cholesterol, and amikacin for 72 hours [13]. However, there is a lack of data on clearance or turnover rates in vivo of sterols in laboratory animal lung surfactant for comparative purposes [1, 8, 14]. In the present study, we measured the clearance rate of radiolabeled cholesterol in vivo in intact rats and examined the extent to which radiolabeled cholesterol was retained in rat lungs after several days to weeks post instillation. 

## 2. Materials and Methods

### 2.1. Instillation of Radiolabeled Cholesterol into Animal Lungs

The lungs of viral antibody-negative female Lewis rats (Harlan Sprague-Dawley, Indianapolis, IN) that weighed between 243 g and 325 g were intratracheally instilled with radiolabeled cholesterol. The material instilled was prepared using [1, 2-^3^H]cholesterol obtained from Amersham (Piscataway, NJ; sp. act. 48 Ci/mmol). Forty mCi in 4 mL of toluene was placed in a 15 mL glass tube, dried under N_2_, and then 1.2 mL of a buffered solution (150 mM NaCl, 1.8 mM CaCl_2_, 25 mM HEPES, pH 7.4, 329 mOsm) was added. After vortex agitation and sonication for 15 minutes, the micellar suspension clarified. 

The rats were mildly anesthetized with 70% CO_2_, then the cholesterol suspension was brought up into a syringe, and 0.15 mL (400 *μ*Ci) were intratracheally instilled into individual rats using a 20-gauge curved stainless steel animal feeding tube (Popper and Sons, New Hyde Park NY) [15, 16]. At different time-points post instillation, rats were sacrificed with halothane, and their lungs were lavaged three times, each time using 1 mL of the HEPES-buffered solution described above. The three bronchoalveolar lavage fluid (BALF) samples were pooled. The postlavage lungs were first perfused via the right atrium to remove blood elements and then excised. Excised lungs were cut into small pieces and homogenized using a Stomacher lab blender 80 (Tekmar, Cincinnati, OH) prior to lipid extraction. Protocols for this study were approved by the Institutional Animal Care and Use Committee and the Radiation Safety Office of the University of Cincinnati.

### 2.2. Extraction of Lipids and Isolation and Analysis of Sterols

Total lipids were extracted for 1 hour at room temperature in chloroform (CHCl_3_) and methanol (MeOH); final proportions of CHCl_3_ : MeOH : BALF or postlavage lung pieces were 1 : 2 : 0.8 (v/v/v) [17]. Particulates were removed from the postlavage lung samples by centrifugation at 1,500 g for 10 minutes. Lipids in extracts of BALF and postlavage lungs were purified by biphasic partitioning [18]; final proportions of CHCl_3_ : MeOH : H_2_O were 2 : 1 : 0.75 (v/v/v). The lower organic phase containing the total lipid fraction was dried under N_2_ and quantified gravimetrically.

Sterols were isolated by thin-layer chromatography (TLC) using glass-backed 250-*μ*m-thick Silica Gel G plates (AnalTech, Newark, DE) and developed in petroleum ether : diethyl  ether : acetic acid (80 : 20 : 1, v/v/v) as the solvent system. Following development, the lipid bands were identified by visualization using I_2_ vapor, and the bands corresponding to free cholesterol (*R*_*f*_ = 0.22), other sterols (*R*_*f*_ = 0.30), and the steryl esters (*R*_*f*_ = 0.85) were scraped off the plates, and radioactivity of these fractions were measured by liquid scintillation spectrometry. Authentic cholesterol, lanosterol, and cholesteryl palmitate were used as standards. Statistical analyses and production of illustrations were performed using Microsoft Excel software.

## 3. Results and Discussion

### 3.1. Total [^3^H]Cholesterol in BALF and Postlavaged Rat Lungs

**Figure 1:**
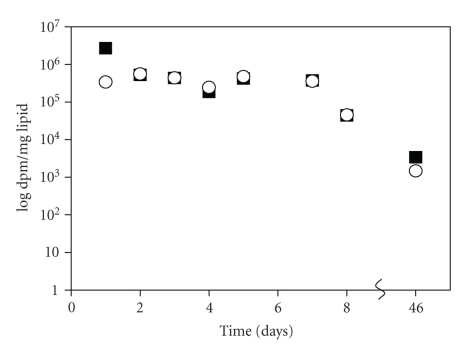
Clearance of radioactivity from [^3^H]cholesterol instilled into rat lungs. Values represent total radioactivities in TLC-isolated bands corresponding to free sterols and steryl esters. BALF (circles) and postlavage lung (squares). Each value represents a single sample from an individual rat.

Under the experimental protocol used in this study, individual animals could not be monitored over time since rats needed to be sacrificed to obtain lungs. Hence, the amount of radioactive cholesterol that had been successfully instilled into the lung alveoli of each rat could not be determined and variations in data would be expected due to individual differences among animals. After different periods following instillation, the sterols in BALF and postlavage lungs of individual animals were found to contain radioactive sterols, indicating that radiolabeled cholesterol reached the lungs of each experimental rat. The radioactivity was higher in lipids extracted from animals sacrificed earlier compared to that in animals sacrificed later (Figure 1). The radioactivity in the TLC fractions corresponding to authentic free cholesterol and steryl esters ranged from 7.9 × 10^3^ dpm to 6.3 × 10^5^ dpm (BALF) and from 6.4 × 10^5^ dpm to 1.7 × 10^7^ dpm (postlavage lungs). The clearance rates of [^3^H]cholesterol from the BALF and postlavage lung were approximately 9% per day and 10% per day, respectively. 

After 46 days post instillation, radioactivity in sterols was still detected in lung samples. At that time, the radioactivity of the free cholesterol TLC fraction was 5,734 dpm (3,066 dpm/mg total lipids) in the BALF and 48,723 dpm (1,372 dpm/mg total lipids) in the postlavage lung. The TLC band corresponding to other sterols was radioactive suggesting metabolism of the instilled cholesterol or that tritium exchange had occurred. The radioactivity in the TLC steryl ester fraction was 375 dpm (201 dpm/mg total lipids) for the BALF, and 979 dpm (28 dpm/mg total lipids) for the postlavage lung. 

### 3.2. Free Cholesterol in BALF and Postlavage Lungs

**Figure 2:**
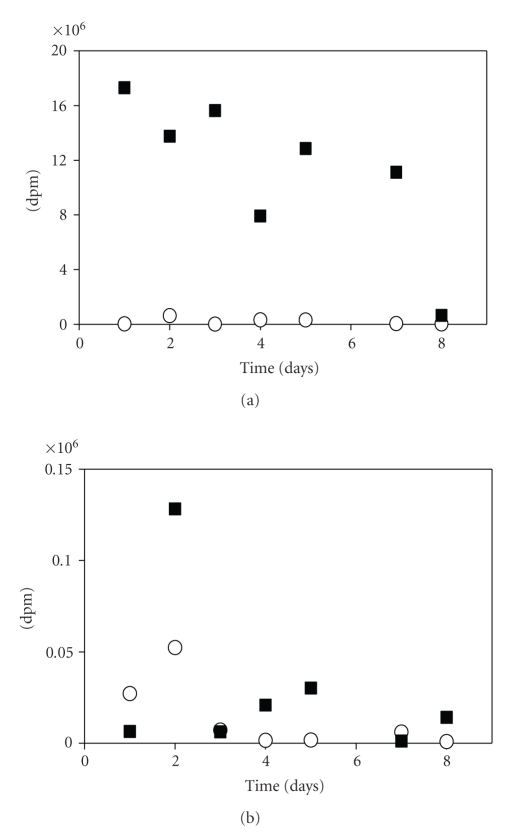
Clearance of radioactivity from [^3^H]cholesterol-instilled rat lungs. Free sterol and the steryl ester fractions were isolated by TLC from BALF (circles) and postlavage lungs (squares). (a) Radioactivity in TLC band corresponding to free cholesterol. (b) Radioactivity in steryl esters.

Since [^3^H]cholesterol was instilled into the trachea, this compound would first be expected to reside in the hyperphase fluid lining the alveoli, which is sampled during bronchoalveolar lavaging. Our data indicate that after 24 hours, the radiolabeled compound must have been readily taken up by pneumocytes since most of the label in the free cholesterol fraction was in the postlavage rat lung tissue compared to the compartment accessible for recovery in lavage fluid (Figure 2(a)). 

### 3.3. Radioactive Steryl Esters in BALF and Postlavaged Rat Lungs

Rat lungs contain very low levels of steryl esters compared to free sterols [19], and in the present study, higher radioactivity was found in free sterols (Figure 2(a)) compared to steryl esters (Figure 2(b)). Radioactivity was detected in all steryl ester samples and ranged from 926 to 52,375 dpm in BALF lipids, and from 1,176 to 128,212 dpm in postlavage lung lipids. It is not known whether the instilled [^3^H]cholesterol is esterified within pneumocytes, such as the type II epithelial cells, within other cells in the lung such as macrophages, or in nonpulmonary tissues of the body.

### 3.4. Comparison with Other Studies on Lung Surfactant Turnover

In experiments performed on rabbits by other investigators, both radiolabeled SP-A and DPPC and other phospholipids were initially cleared from the lungs at exponential rates and then at a slower linear rate [1, 8]. Since SP-A disappeared at a faster rate than DPPC, this indicated separate pathways for secretion of these two major components of lung surfactant [1, 9]. In the present study, the first measurements were taken 1 day post instillation. Together with data in the literature on rabbits [10], we tentatively conclude that our results reflect the linear clearance rate of ~10% per day that may occur following an initial rise and steep decrease seen at earlier times in the rabbit lung [10] and human [13] studies. Hence only low percentages of instilled radioactivity were recovered in free cholesterol and steryl esters present in BALF and postlavaged lungs after a period of days (Figure 1) and weeks.

The formation of cholesterol esters from instilled radiolabeled free cholesterol can occur rapidly [20]. Using an isolated perfused rat lung preparation, Hayball and Nicholas [20] reported that an alveolar macrophage-enriched fraction of BALF contained higher levels of the cholesterol esters compared to the surfactant-enriched fraction of BALF. However, they reported that radiolabeled cholesterol esters were not formed in vitro when radiolabeled free cholesterol was incubated with these cell fractions. Isolated alveolar epithelial cells or postlavage lungs were not tested in that study. Thus, it is likely that at least some of the radioactive cholesteryl esters detected in the present study might have formed in the lung in vivo. However, the data in an earlier report [20] and those in the present study do not rule out the possibility that cholesterol is translocated to other tissues of the rat, esterified or otherwise metabolized, and then translocated back to the lung. 

### 3.5. Translocation of Sterols in Animals

In the present in vivo study, radioactivity was also detected in liver, mesenteric fat, feces, heart, blood, and urine in rats from day 1 to day 8 post instillation, indicating that cholesterol is readily translocated to different organs and tissues of the rat. The total radioactivity recovered from lungs after days or weeks post instillation must strongly reflect the elimination of radiolabeled cholesterol by other organs in the animals. Cholesterol would be expected to be readily imported into the rat lung since it has been reported that de novo sterol synthesis does not occur in this organ [21] and 3-hydroxy-3-methylglutaryl Coenzyme A (HMG-CoA) reductase activity was undetectable or at only very low levels in rat lung tissue [22]. 

## 4. Conclusions

Data obtained in this study showed that radioactivity from cholesterol instilled in vivo into rat lungs was detected in both BALF and postlavage lung lipids after several weeks and that the clearance rate over the initial period of a week was calculated at approximately 10% per day. Although the radioactive sterols measured in the lungs after instillation of [^3^H]cholesterol are very likely influenced by elimination and metabolism by other organs in the rat, the results of this study showed the persistence of high levels of radiolabeled cholesterol in the rat lung. This is the first study in which cholesterol clearance was performed in vivo over days and weeks in intact laboratory animals. The data reported here are consistent with other evidences of recycling of lung surfactant components in the mammalian lung.
